# Human Kinetoplastid Protozoan Infections: Where Are We Going Next?

**DOI:** 10.3389/fimmu.2018.01493

**Published:** 2018-07-25

**Authors:** Alessandra Almeida Filardy, Kamila Guimarães-Pinto, Marise Pinheiro Nunes, Ketiuce Zukeram, Lara Fliess, Ludimila Pereira, Danielle Oliveira Nascimento, Luciana Conde, Alexandre Morrot

**Affiliations:** ^1^Department of Immunology, Paulo de Góes Microbiology Institute, Federal University of Rio de Janeiro, Rio de Janeiro, Brazil; ^2^Immunoparasitology Laboratory, Oswaldo Cruz Foundation, Oswaldo Cruz Institute, FIOCRUZ, Rio de Janeiro, Brazil; ^3^Tuberculosis Research Center, Faculty of Medicine, Federal University of Rio de Janeiro, Rio de Janeiro, Brazil

**Keywords:** parasitic infection, kinetoplastid protozoans, *Trypanosoma cruzi*, *Leishmania*, *Trypanosoma brucei*

## Abstract

Kinetoplastida trypanosomatidae microorganisms are protozoan parasites exhibiting a developmental stage in the gut of insect vectors and tissues of vertebrate hosts. During the vertebrate infective stages, these parasites alter the differential expression of virulence genes, modifying their biological and antigenic properties in order to subvert the host protective immune responses and establish a persistent infection. One of the hallmarks of kinetoplastid parasites is their evasion mechanisms from host immunity, leading to disease chronification. The diseases caused by kinetoplastid parasites are neglected by the global expenditures in research and development, affecting millions of individuals in the low and middle-income countries located mainly in the tropical and subtropical regions. However, investments made by public and private initiatives have over the past decade leveraged important lines of intervention that if well-integrated to health care programs will likely accelerate disease control initiatives. This review summarizes recent advances in public health care principles, including new drug discoveries and their rational use with chemotherapeutic vaccines, and the implementation of control efforts to spatially mapping the kinetoplastid infections through monitoring of infected individuals in epidemic areas. These approaches should bring us the means to track genetic variation of parasites and drug resistance, integrating this knowledge into effective stewardship programs to prevent vector-borne kinetoplastid infections in areas at risk of disease spreading.

## Introduction

Protozoan infections are one of the most devastating causes of human death worldwide. These infections are caused by protozoan parasites, microorganisms originally classified in the Kingdom Protozoa, which comprises a diverse group of unicellular eukaryotes (1). Although the majority of the protozoan exists as free-living microorganisms in different aquatic and humid environments, there are many species living in association with host organisms, causing severe human diseases (1, 2). This is the case of Kinetoplastid parasites, a group of flagellated protozoans that parasitize most plant and animal species; and cause human diseases with public health threats and social-economic effects (3).

**Figure 1:**
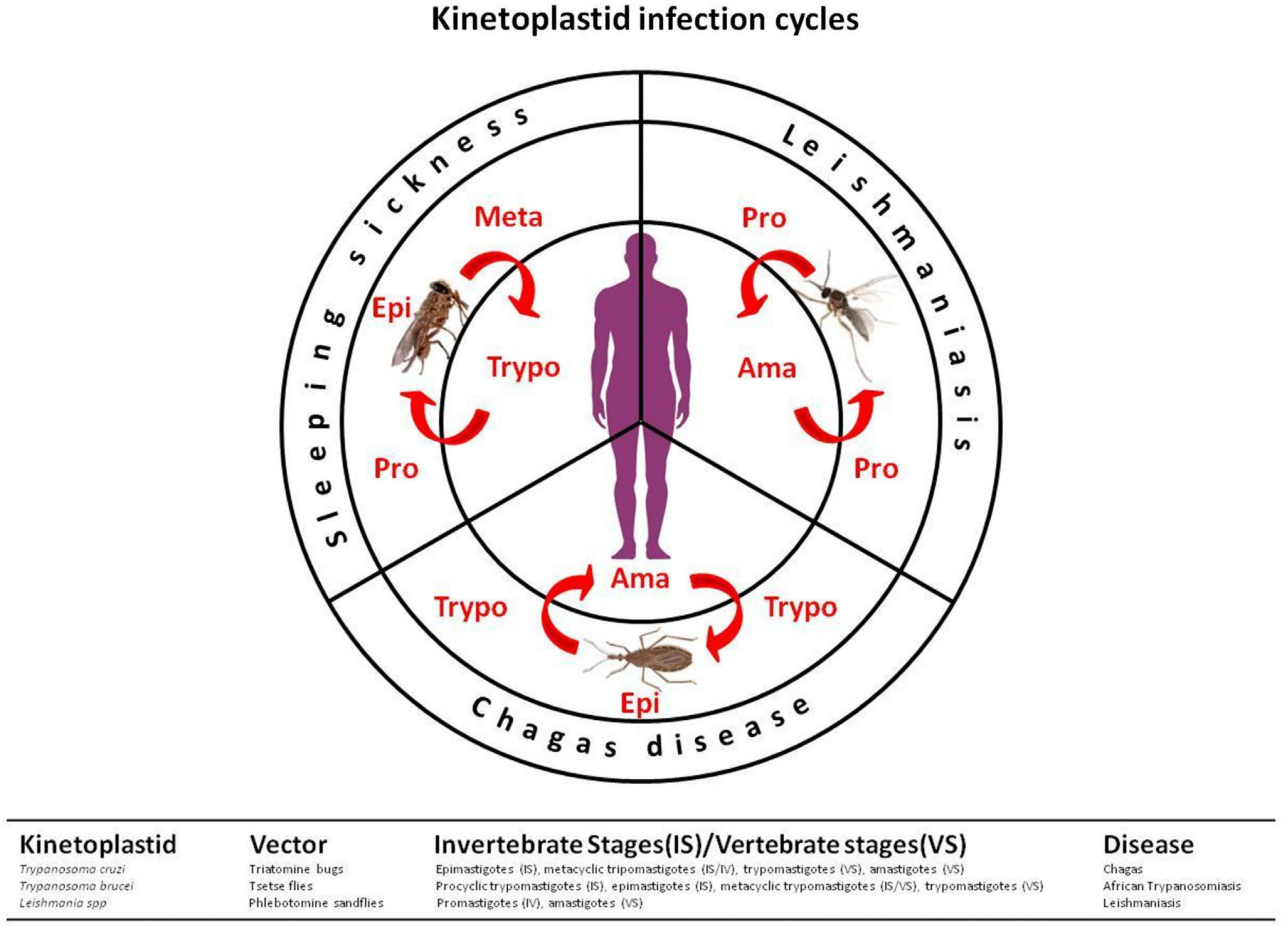
Kinetoplastid transmission cycles. The life cycles of kinetoplastid protozoan parasites show development stages in both invertebrate and vertebrate hosts. In leishmaniasis, promastigotes (flagellate and mobile forms) are inoculated into the skin along with the saliva of phlebotomine vectors (Diptera: Psychodidae). In the vertebrate hosts, they are maintained inside phagocytic cells under the proliferative form, amastigotes. When ingested by insect vectors, the amastigote forms are transformed into promastigotes which multiply in the intestinal tissue, then migrate as metacyclic promastigotes into the stomodal valve to be later injected into the skin during blood meal. In Chagas’ disease, the etiologic agent *Trypanosoma cruzi* is transmitted to vertebrate hosts as a metacyclic trypomastigote forms by infected triatomine bugs (Triatominae: Reduviidae) during blood feeding. Once in the vertebrate, trypomastigotes differentiate into intracellular amastigote forms. These proliferative stages multiply by binary division, and then differentiate into trypomastigotes, which are released into the bloodstream. When the triatomine bug takes a blood meal from an infected vertebrate host containing circulating parasites, the ingested trypomastigotes forms to differentiate into epimastigotes in the medium intestine of the vector, multiplying by binary division, after which they differentiate into infective metacyclic trypomastigote forms. In sleep sickness or human African trypanosomiasis, the parasite *Trypanosoma brucei* is transmitted by the bite of the tsetse fly (Glossinidae: Glossina). The parasite exists in the saliva of the invertebrate vector and is injected when the insect feeds on human blood. Unlike *Trypanosoma cruzi*, trypomastigotes of *T. brucei* do not invade host cells and, therefore, does not differentiate into intracellular amastigote forms. Instead, *T. brucei* parasites multiply as trypomastigotes in the blood of infected vertebrate host. The parasite cycle continues when a new vector feeds on a contaminated individual. In the invertebrate vector, the parasites differentiate into proliferative epimastigotes forms, invading the insect salivary glands to continue the cycle.

The kinetoplastids are a monophyletic group related to the euglenids. These microorganisms are distinguished from other protozoan groups mainly by the presence of kinetoplasts, a granule that contains “kDNA,” a DNA located in the mitochondria, associated with the base of the flagella (3). Three distinct kinetoplastids cause human disease: *Trypanosoma brucei* [human African trypanosomiasis (HAT) or sleeping sickness], *Trypanosoma cruzi* (Chagas disease), and *Leishmania* spp. (leishmaniasis) (3, 4), which are still recognized as neglected tropical diseases (NTDs) by the World Health Organization. These two genera of parasites are found in the blood and/or tissues of infected humans and are transmitted by arthropod vectors (Figure 1) (5).

**Figure 2:**
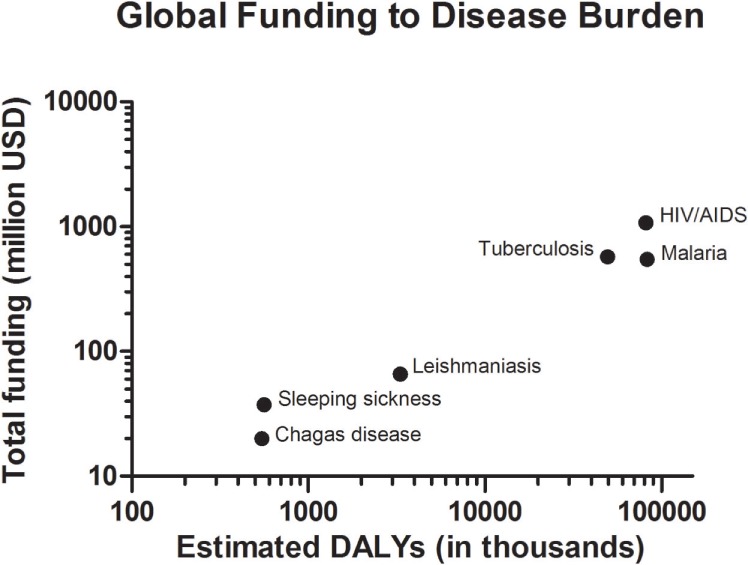
Overall funding for research and development investment correlated with disability-adjusted life years, estimated from the Global Burden of Disease Study.

Although the advances in the development of drug therapies and vector control agents against kinetoplastid diseases (6), new strategies are required for global elimination of epidemies. The main limiting efforts for this accomplishment obviously relies on the global investments in R&D for these NTDs when compared to other diseases with higher levels of financial support, such as malaria, tuberculosis, and HIV, known as “big three” (7). The analysis of the global sums of expenditures for each of these diseases, and their correlations with the social impact indexes on public health indicates a correlation that goes far beyond the political question (8). Such correlation is proportional to the severity of each disease (Figure 2), as to the metric of the disability-adjusted life year (DALY), an index calculated as the sum of the years of life lost due to premature mortality of patients and the years lost due to disability for people living with the illness (9).

In spite of the low priority of the investments received, studies in the past decades have pointed out the paths for developing effective actions to control human kinetoplastid protozoan infections through a better understanding of the pathogen–vertebrate host interactions during their life cycles, disease pathogenesis in the hosts, and methods that allow the diagnosis, even in the acute phase of the infection (3, 4). This has provided a better opportunity to prospect new therapeutic targets, more effective drug development approaches, and promising vaccines that, together, may combat these diseases more robustly in the near future (5).

## Trends in Drug Development for Kinetoplastid Infections

Treatment approaches based on drug therapies in kinetoplastid infections are scarce and have been considered to be highly toxic (10). However, interest in research and exploration of new approaches has given a sign of change. Since kinetoplastid infections lead to chronic persistent diseases, it is critical that drug therapy interventions would be focused on the acute phase, with hopes of achieving complete eradication of disease transmission. To that end, it is necessary to establish technical procedures that allow early diagnosis of acute kinetoplastid infection (5). This intervention approach aims to act at a stage of infection in which pathogen has not yet established itself and is present with a low parasitism burden and few down modulatory mechanisms in place over the host immune responses (1, 11, 12).

In addition, a rapid and efficient intervention during acute phase of the infection, when the host is still under low tissue parasitism, decreases the chances of acquiring drug resistance (13, 14). In Chagas disease, benznidazole and nifurtimox have long been the only clinical treatment options for infection (15). However, progress has been made in the research of new promising drugs against the disease. E1224, a pro-drug of ravuconazole, has shown some efficiency in combating the disease (16). The results of the first phase 2 clinical trial in Bolivia, conducted by drugs for neglected diseases initiative, have shown satisfactory protective results for E1224. This drug was effective in controlling *T. cruzi* parasitism in infected patients. Furthermore, its use in combination with benzanidazole is more effective as compared to monotheraphy-based protocols (16).

To treat HAT, five drugs have been approved: pentamidine, melarsoprol, eflornithine, suramin, and nifurtimox (17). Pentamidine and suramin are used in monotherapies in the early stage of *T.b. gambiense* and *T.b. rhodesiense* infections, whereas melarsoprol is used for the second stage of the disease (18, 19). Nifurtimox has been used since 2009 in combination with eflornithine, mostly in the second stage of *T.b. gambiense* infection. The combination therapy protocols for these two drugs have been improved, although there are still practical restrictions to their potential use in large-scale applications (20). Currently, new drugs designed to improve patient care are being considered to meet current elimination targets. Most of them have been optimized to undergo clinical trials (17).

Fexinidazole and oxaborole SCYX-7158, are already being studied in clinical evaluation as oral therapies in phase IIb/III and phase I trials, respectively (21, 22). Other important R&D studies have focused on the virulence responses of the parasite. The cell surface of African trypanosomes (*T. brucei* spp.) is covered by a dense coat of glycoconjugates that play important roles in the evasion of host immune responses (23). New strategies in drug discovery against HAT aim the development of specific carbohydrate-binding agents capable of inhibiting the action of glycosyltransferases and glycosidases of the parasite, thus altering the nature of the parasite’s cell-surface glycans as a treatment target for sleeping sickness (24).

In leishmaniasis, a notable progress in the disease treatment has been made in the preparation of new formulations of amphotericin B, using liposome carriers (25). In addition, new drugs have been extensively studied in clinical trials, although the effects of HIV co-infection in endemic areas contribute to drug unresponsiveness during therapies (26). The use of paromomycin, an aminoglycoside class antibiotic, has also been shown to be efficient at low cost as a first-line drug (27). Alternatively, studies have suggested the effective action of drugs that act on a broad spectrum against *Leishmania* parasites. This is the case of miltefosine, a drug that is also used in the treatment of dogs with leishmaniasis in Brazil (28). The miltefosine has an inhibitory action on several biological pathways of the parasite, such as cytochrome C oxidase, synthesis of phosphatidylcholine, and disruption of parasite Ca^2+^ homeostasis (29–31).

In addition, drug discovery researches for kinetoplastids have benefited from investments made in the field of protozoan genomics. This is clearly seen in the use of data mining, annotation, and analysis of *Leishmania* parasite genome that has lead to the creation of LeishCyc database (32). This systematic approach will allow a complete mapping of *Leishmania* transcriptomics, proteomics, and metabolomics enabling the development of new compounds that can be used in high-throughput screening approaches to search selective drugs against *Leishmania* parasites (32). In fact omics-based analyses have facilitated the broadening of R&D researches in the field of drug development. Lipidomics analyses have yielded the characterization of lipid structures, protein lipidation pathways in post-translational modifications, and their putative functions for kinetoplastid parasites (33, 34). This knowledge will allow the search for new classes of anti-parasitic pharmaceuticals.

## Research and Development of New Vaccines Against Kinetoplastid Parasites

The major challenge in the preventive control of kinetoplastid infections and other neglected tropic infectious diseases is undoubtedly the implementation of low-cost vaccines for public health programs in affected countries, most of them were low- and middle-income countries (5, 35). This accomplishment would enable to structure intervention actions in health care at the level of mass treatment programs, thus avoiding disease re-emergence. To reach this ideal scenario, there are serious social-political obstacles to overcome in order to ensure the development of clinical tests. The most incisive limitation is unquestionably economic, resulting from a scarce financial incentives and market failures in view of the geopolitical areas where epidemics occur (35). Such constraints are likely to arouse less interest from financial and pharmaceutical institutions in the market development strategies for vaccines against NTDs in general.

There are no licensed vaccines for kinetoplastid infections yet, reinforcing the need for their development, particularly in countries where they are epidemic. Most of the ongoing vaccine studies have been conducted at the level of basic research (5). Particularly, recent advances in the construction of manipulative parasites through genome engineering using CRISPR/Cas9 and Cre recombinase have been dedicated to reprogramming their genome, allowing the identification of regulatory genes associated with the cell fates in the host–parasite interplay (36). These critical virulence genes represent good candidates to be studied as vaccine targets.

Interesting, some kinetoplastid vaccines undergoing clinical trials are promising, since they are cost-effective and show long-term protection against both cutaneous and visceral leishmaniasis. This is the case of vaccines developed by the Infectious Diseases Research Institute, which has included protective *Leishmania* antigen epitopes in its vaccine formulations used in clinical trials (37, 38). Another prominent proposal comes from initiative studies in cooperation with National Institutes of Health. They present a more elaborate vaccine formulation, including protective recombinant *Leishmania* antigens in combination with sand fly salivary gland antigens, capable of inducing a more robust host immune response, considering vector–host interactions in the transmission of *Leishmania* parasites (39).

In the case of Chagas disease, initial studies have pointed out potential antigens in experimental mice models capable of inducing host protective immune responses. They were also effective in reducing cardiac parasite loads and disease pathology, increasing host survival indices. Those studies have proposed the use of Tc24 (calcium binding protein associated with flagellar pocket of *T. cruzi*) and TSA-1 (Trypomastigote surface antigen-1) by a global consortium (40–42). These are the first candidates to be used in vaccine formulations to prevent Chagas disease. Furthermore, the use of vaccines as a therapeutic approach to treat chagasic patients has also been proposed (42).

## Key Challenges to Kinetoplastid Parasite Control: Integrating New Approaches into Eradication Strategies

To eradicate kinetoplastid infections in humans, we must gain a better understanding of variants of pathogens and their vectors, the transmission models among their hosts, and efficacious preventive vaccine approaches, to understand and design strategies to intervene in the real dynamics of disease spreading in epidemic areas (43). To achieve that, diagnostic tests capable of detecting infections in the acute phase, and accurate analysis of drug resistance in the parasite spreading are essential to trace a better correlation between variants of pathogens and the clinical spectrum of the disease. Another important step of intervention in the transmission cycles of vector diseases is regarding the application of health care models based on geographic information system (GIS) projects and technologies (44). By using this system, we will be able to follow the infection cycle in terms of spatial data, geographical and geodatabases, which allows us to create mapping models to predict potential areas of risk for transmission of vector-borne diseases (45).

**Figure 3:**
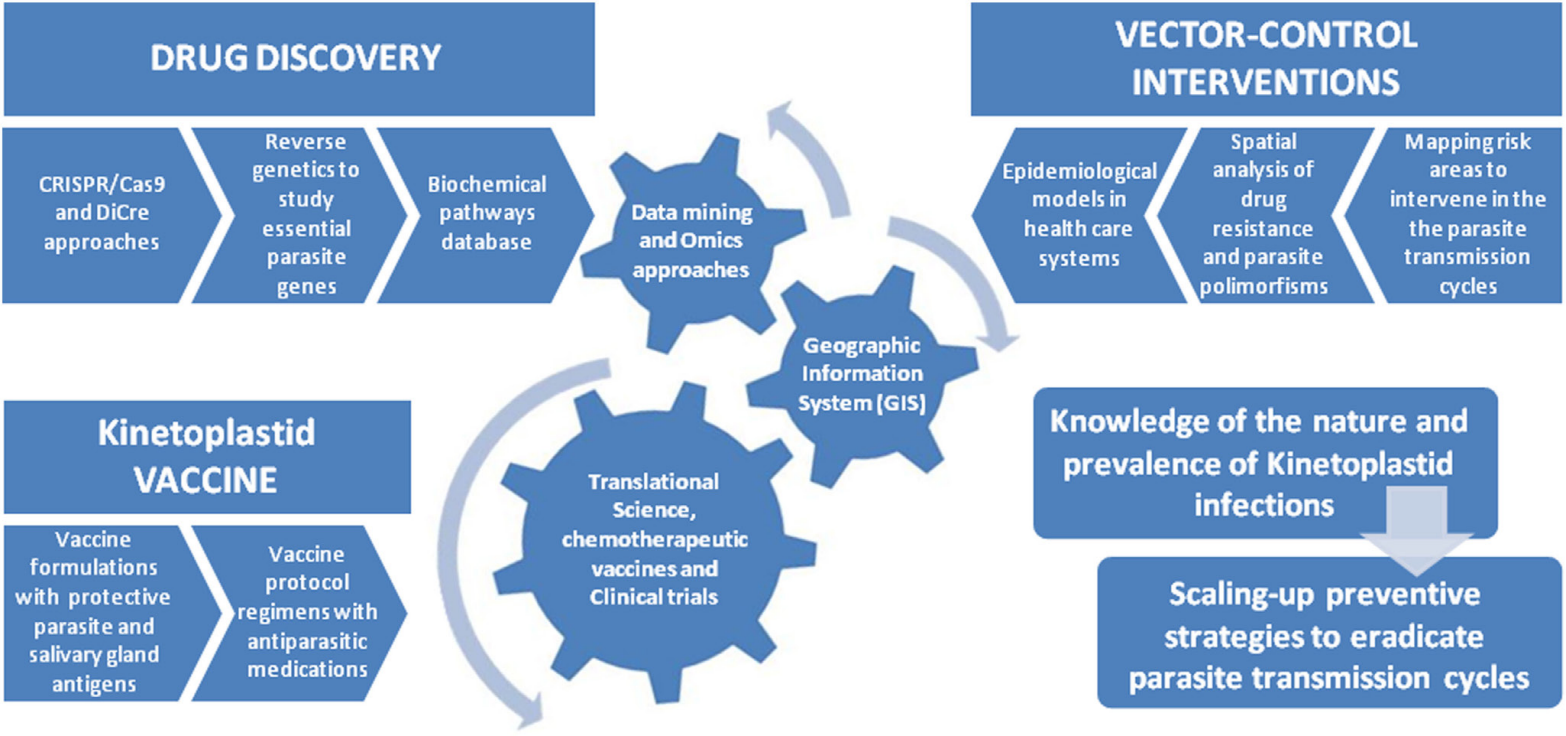
Integrating new approaches into control strategies for kinetoplastid infections. In the past decade, genetic sequencing efforts and manipulation of trypanosomatid genomes have elucidated the characterization of virulence factors and parasite biochemical pathways involved in the pathogenesis disease. These advances allowed the design of new drug targets and therapeutic vaccines capable of reducing the parasitic burden thus controlling the infection and its clinical symptoms. The integration of these tools with epidemiological interventions into public health programs should yield substantial gains in controlling the transmission cycles of these vector-borne diseases. In this line, important studies in the field of vector control interventions have defined a multi-parametric analysis using geographic information system technologies to monitor spatial analysis of drug resistance and parasite polymorphisms to delineate epidemic-prone areas. Those studies have offered a powerful platform for prospecting and development of tools and technologies capable of effectively defining elimination programs in the control and eradication of kinetoplastid diseases.

The GIS technology is able to compile multi-analysis related to public health data collected from endemic urban and wild areas, including analyses of disease dispersal, genetic variation of parasites, vector and host habitats, and their relationship with microclimates in affected areas. These metadata analyses can be correlated with geographic positioning system to establish interventions of potential infections, thus allowing for a rational use of medications to prevent the emergence of drug resistance in the context of disease surveillance (44, 45). Nowadays, diverse stochastic models are used as tools for estimating probability distributions of potential outcomes from the understanding of environmental and ecological networks involved in the cycles of vector-borne diseases. These geographic models have allowed disseminating critical information concerning public health surveillance for endemic areas (Figure 3).

In kinetoplastid infections, spatial clustering using GIS technology when applied with accurate diagnostic test, analysis of the phylogenetic distribution of *Leishmania* spp. together with surveillance has been demonstrated to be of great value in predicting areas of risk. Epidemiological analysis of the transmission cycles in Europe, and in recent kala-azar outbreaks in Nepal, Kenya, and Brazil, have helped to precisely define that the priority of intervention is in the vector control with the use of insecticides, in order to block the transmission cycle of disease in potential risk areas, identified to be priority to reduce the spread of infection (46–49).

## Concluding Remarks

Neglected tropical diseases caused by kinetoplastid protozoan parasites are considered endemic in lower-middle-income economies, although in many countries they are in process of epidemiological containment and elimination. However, the eradication of these diseases is a complex issue that must involve health management policies, coordinated among the affected countries, such as the development of public policies to combat parasitic diseases and improvement of social conditions. This will only be possible through the cooperation of local governments in a participatory search for bilateral relations between public and private interests capable of fostering translational studies that guarantee the access of the population to appropriate treatments. The development of accurate diagnosis capable of identifying these diseases in the acute phase of infection, as well as the identification of parasite polymorphisms and variants of drug resistance, together with application of health care models based on GIS technologies will enable both preventive and therapeutic actions based on new generations of drugs and vaccines currently developed for these neglected diseases.

## Author Contributions

AM conceived the manuscript. AAF and AM wrote the manuscript. AAF, KG-P, MPN, KZ, LF, LP, DON, LC, and AM participated in the preparation of the manuscript.

## Conflict of Interest Statement

The authors declare that the research was conducted in the absence of any commercial or financial relationships that could be construed as a potential conflict of interest.
